# Virtual Systems Pharmacology (ViSP) software for simulation from mechanistic systems-level models

**DOI:** 10.3389/fphar.2014.00232

**Published:** 2014-10-22

**Authors:** Sergey Ermakov, Peter Forster, Jyotsna Pagidala, Marko Miladinov, Albert Wang, Rebecca Baillie, Derek Bartlett, Mike Reed, Tarek A. Leil

**Affiliations:** ^1^Exploratory Clinical and Translational Research, Bristol-Myers SquibbPrinceton, NJ, USA; ^2^Forster Solutions, LLCWilmington, DE, USA; ^3^Research IT and Automation, Bristol-Myers SquibbPrinceton, NJ, USA; ^4^Rosa & Co. LLCSan Carlos, CA, USA

**Keywords:** quantitative systems pharmacology, system-level mechanistic models, virtual patient, model development software, simulation experiment, metabolic diseases model

## Abstract

Multiple software programs are available for designing and running large scale system-level pharmacology models used in the drug development process. Depending on the problem, scientists may be forced to use several modeling tools that could increase model development time, IT costs and so on. Therefore, it is desirable to have a single platform that allows setting up and running large-scale simulations for the models that have been developed with different modeling tools. We developed a workflow and a software platform in which a model file is compiled into a self-contained executable that is no longer dependent on the software that was used to create the model. At the same time the full model specifics is preserved by presenting all model parameters as input parameters for the executable. This platform was implemented as a model agnostic, therapeutic area agnostic and web-based application with a database back-end that can be used to configure, manage and execute large-scale simulations for multiple models by multiple users. The user interface is designed to be easily configurable to reflect the specifics of the model and the user's particular needs and the back-end database has been implemented to store and manage all aspects of the systems, such as Models, Virtual Patients, User Interface Settings, and Results. The platform can be adapted and deployed on an existing cluster or cloud computing environment. Its use was demonstrated with a metabolic disease systems pharmacology model that simulates the effects of two antidiabetic drugs, metformin and fasiglifam, in type 2 diabetes mellitus patients.

## Introduction

In recent years pharmaceutical R&D has seen an increase in the development and application of mechanistic, systems-level models to inform decision making. These models are better at describing the disease biology and drug pharmacology than the more traditional and empirical pharmacokinetic/pharmacodynamic (PK/PD) models (Lalonde et al., 2007; Milligan et al., 2013; Visser et al., 2013). They are typically called quantitative systems pharmacology (QSP) models to distinguish them from other systems biology models that do not incorporate drug pharmacology or pharmacokinetics and often do not account for the biology of disease and its progression. With a detailed representation of physiology and pharmacology QSP models include a significantly larger number of equations and parameters compared to what is normally seen in traditional PK/PD models (Mager et al., 2003; Danhof et al., 2007). Despite the challenges of accurately determining all of the model parameters, QSP models can nevertheless be very informative by allowing the generation of quantitative hypotheses about the efficacy and/or safety of drugs prior to testing them in humans, or when testing in new patient populations (De Graaf et al., 2009; Kuepfer et al., 2012). Examples of mechanistic system-level models include, the first attempt to mathematically model the circulatory system in the human body (Guyton et al., 1972) and more recently, the HumMod model (Hester et al., 2011), and models of glucose homeostasis (Schaller et al., 2013), rheumatoid arthritis (Rullmann et al., 2005), hypertension (Hallow et al., 2014), and drug induced liver injury (DILI) (Shoda et al., 2014). A recent review by Schmidt et al. (2013) describes the process of how these models can be built and used.

With our incomplete knowledge of disease biology, QSP models can be used to make and test assumptions about the intrinsic variability in biological pathways. Because of the deterministic nature of the current approaches to the development of QSP models, it is expected that one set of initial conditions will normally produce a single set of outcomes with a unique solution trajectory from its initial state to the final one. In order to represent variability observed in a given population, multiple simulations with different initial conditions must be generated, each simulation implementing a virtual experiment or a virtual patient. Combined, the results from a sufficient number of simulations will provide estimates on the expected degree of variability in a population of patients. However, this comes at a price, by involving a large number of simulations and a large number of varied parameters the modeling process becomes computationally intensive. This challenge can be efficiently tackled only by employing the state of the art high-performance computing technology.

Likewise, the creation of large QSP models is not a trivial task; it requires the use of sophisticated and specialized software applications. Available tools range from complex sets of distributed software packages connected through a common portal to smaller yet versatile software programs capable of producing detailed mechanistic models. Examples of the former include the Garuda Alliance (Ghosh et al., 2011) and Physiome project (Thomas et al., 2008; Randall Thomas, 2009), while examples of the latter are JDesigner (Vallabhajosyula and Sauro, 2007), Entelos PhysioLab (Shoda et al., 2010), Mathworks SimBiology (MathWorks)^1^, Bayer's PK-Sim and MoBi (Eissing et al., 2011), and ISB's DBSolve Optimum (Gizzatkulov et al., 2010). For a given problem the choice of a proper modeling tool could become a difficult task by itself. In addition to purely scientific considerations dictated by the scope of the model, the software should meet multiple criteria to be considered optimal: an intuitive user interface, numerous differential equation solvers and library functions, a convenient way of storing and handling large number of parameters, ease of setting up multiple simulations and executing them in parallel, multiple-format import-export capabilities, reasonable cost and technical support, and an existing base of trained users. In this paper we present a simple and user-friendly Virtual Systems Pharmacology (ViSP) platform designed to quickly set up, run, and handle multiple simulation tasks in a flexible and scalable hardware/software environment. The platform is neither model nor software specific and can utilize existing cluster or cloud computing infrastructure for large-scale simulations. The ViSP platform was successfully used with a Metabolic Diseases Systems Pharmacology (MDSP) model to simulate multiple antidiabetic therapies in healthy and Type 2 diabetes mellitus (T2DM) patients.

## Methods

In order to create flexible and versatile QSP software for setting up and running large scale simulations the following requirements were formulated:

1. Handle diverse systems pharmacology models designed by different software packages.
2. Be independent of the specifics of any given model and place as few general requirements as possible on the model (e.g., not depend on the number of parameters, actual parameter values or their names, etc.).
3. Enable a flexible computational environment/hardware choice to run simulations (cluster, cloud, or desktop).
4. Provide an intuitive user interface (UI) that is easily configurable without updating the software code to accommodate specifics of a particular model and the input parameters.
5. Should serve as collaboration software accessible throughout the company network. Offer differentiated access to models and projects, based on set user privileges.
6. Provide means of storing and handling large modeling projects.
7. Have low deployment and maintenance costs.

**Figure 1:**
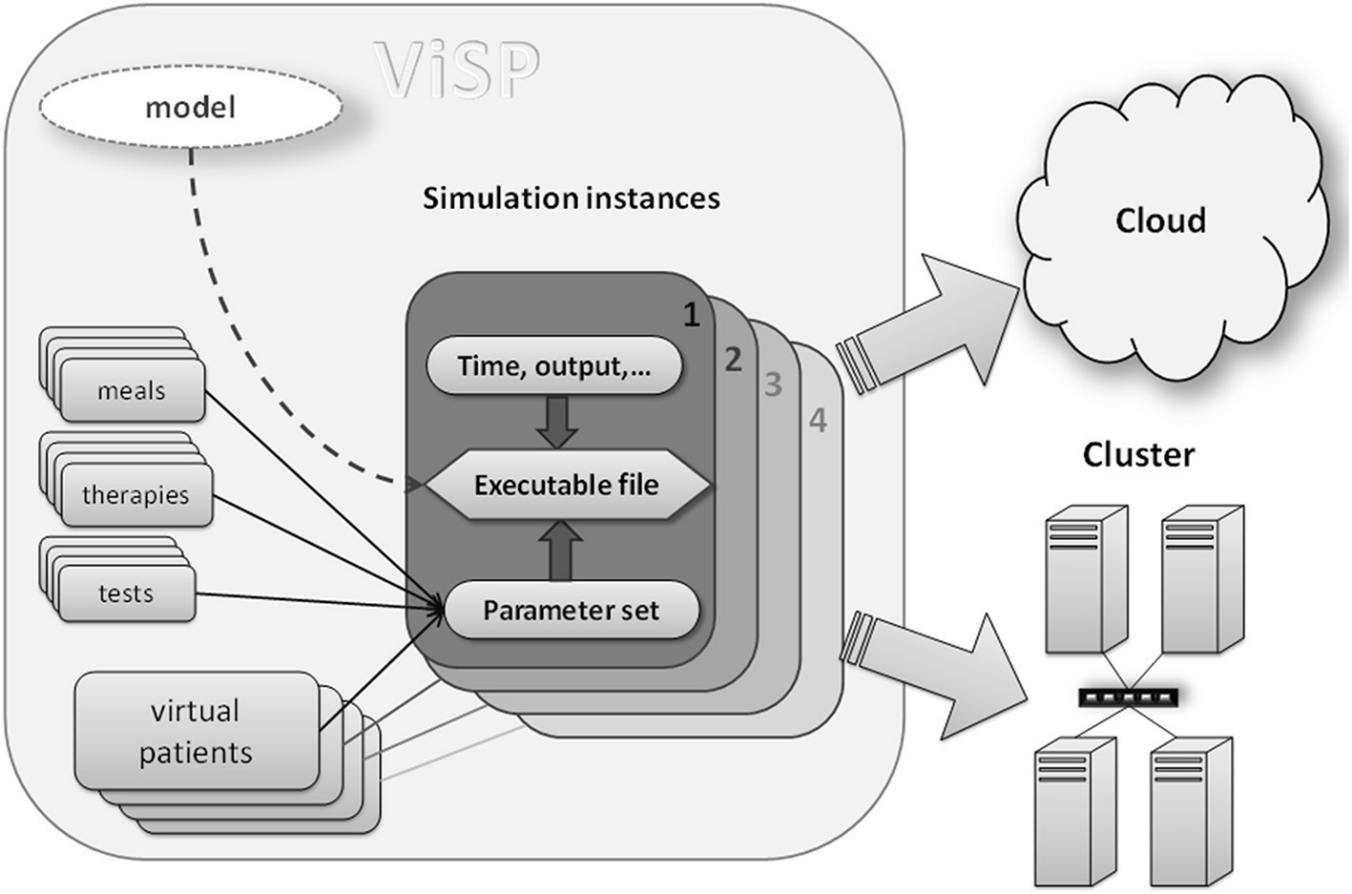
**Components of a simulation: compiled model file (executable) together with input data (e.g., virtual patients, therapies, simulation time) represent a unique simulation instance that can be dispatched to either cluster computer or to a cloud**. Input data provide a mechanism of customizing each particular simulation. Executable file should be compiled for a target operating system (Linux, Unix, Windows) that runs on cluster nodes or a cloud.

The main innovation that enables the first three requirements is to separate the process of constructing a QSP model from the process of running simulations originated by that model. During the design step the model is created and then saved in what could be software proprietary file format. Afterwards it is the operating system (OS) that runs simulations, a process that could be implemented as software independent. One way to achieve this is to compile the model file(s) and convert into an executable code in which high-level model instructions are translated into low-level machine commands. Features proprietary to the modeling software will be removed during this translation and the executable will rely only on OS instructions. In order to keep the simulation process maximally flexible all model parameters need to be external to the executable file (i.e., their values not being hard-coded into the model). This means all model parameters should be presented as input parameters. Once input values are provided, together with the executable they will define a unique simulation task. Multiple simulation instances can be created by combining different input parameter values with executables, which can be run on a grid of processors in a cluster, in a cloud, or in a mixed environment (see Figure 1). As soon as the executable is compiled for the OS which runs the hardware, all computational resources integrated by the grid management software will be available for simulations.

Another important concept of the QSP software architecture is making it a web-based client-server application with a relational database back-end. With many advantages emphasized below it helps to address the requirements formulated in points 4 through 7. For example, a web-based platform is inexpensive to deploy and maintain, since it does not require installing software on every computer and web browsers are now omnipresent. Modifications and upgrades to the software could be done on the server side with no user intervention, thus reducing IT cost and time. Web-based software is easily accessible over networks inside or outside the company by multiple users whose access privileges can be regulated by IT departments. More advantages offered by web-based architecture come from the rich selection of software tools available for UI, front, and back end programming. Additionally, the database allows for a robust, reliable, dynamic, structured and complex relational data model where items such as models, users, virtual patients, project information, UI configuration settings, simulation inputs and results can be stored, managed and displayed by the UI components or other server-side modules.

Since QSP software is required to be capable of handling multiple models, its UI needs to be dynamic and configurable. This means the UI should expose model parameters in the way specific to each particular model. Also, some parameters should be immediately available via UI while other should be hidden. The latter could be necessary because physiological models often have hundreds of parameters, of which only a relatively small subset may be of interest for performing simulations. Such flexibility could be achieved by dividing all model parameters into meaningful groups, e.g., parameters that describe caloric value and composition of meals consumed by patients, or parameters specifying drug regimen, and so on (see Figure 1). Then, only the groups that are of interest will be selected during the UI configuration process; they will show up as sub-sections in the UI with specific parameters inside. An example of such an interface is given in the Results section.

Grouping provides additional benefits for handling and storing parameter values in a structured way. For instance, the same parameter group may get assigned different value sets corresponding to different individuals, here called virtual patients. Similar manipulations can be done with groups describing therapies, meals, and so on. Each set of values can be given a name and stored in the database with options for search and reuse. Once a sufficient number of such value sets is accumulated in the database, the end user's task of setting up simulations will be reduced to simply finding and selecting appropriate value sets. Again, the use of relational database enables and empowers this process, making it another key concept implemented in the QSP software architecture. In addition to parameter value sets, practically all other information about the model, UI configuration, and simulation results is stored in the database that is searchable and that preserves the relationships between these pieces of information.

**Figure 2:**
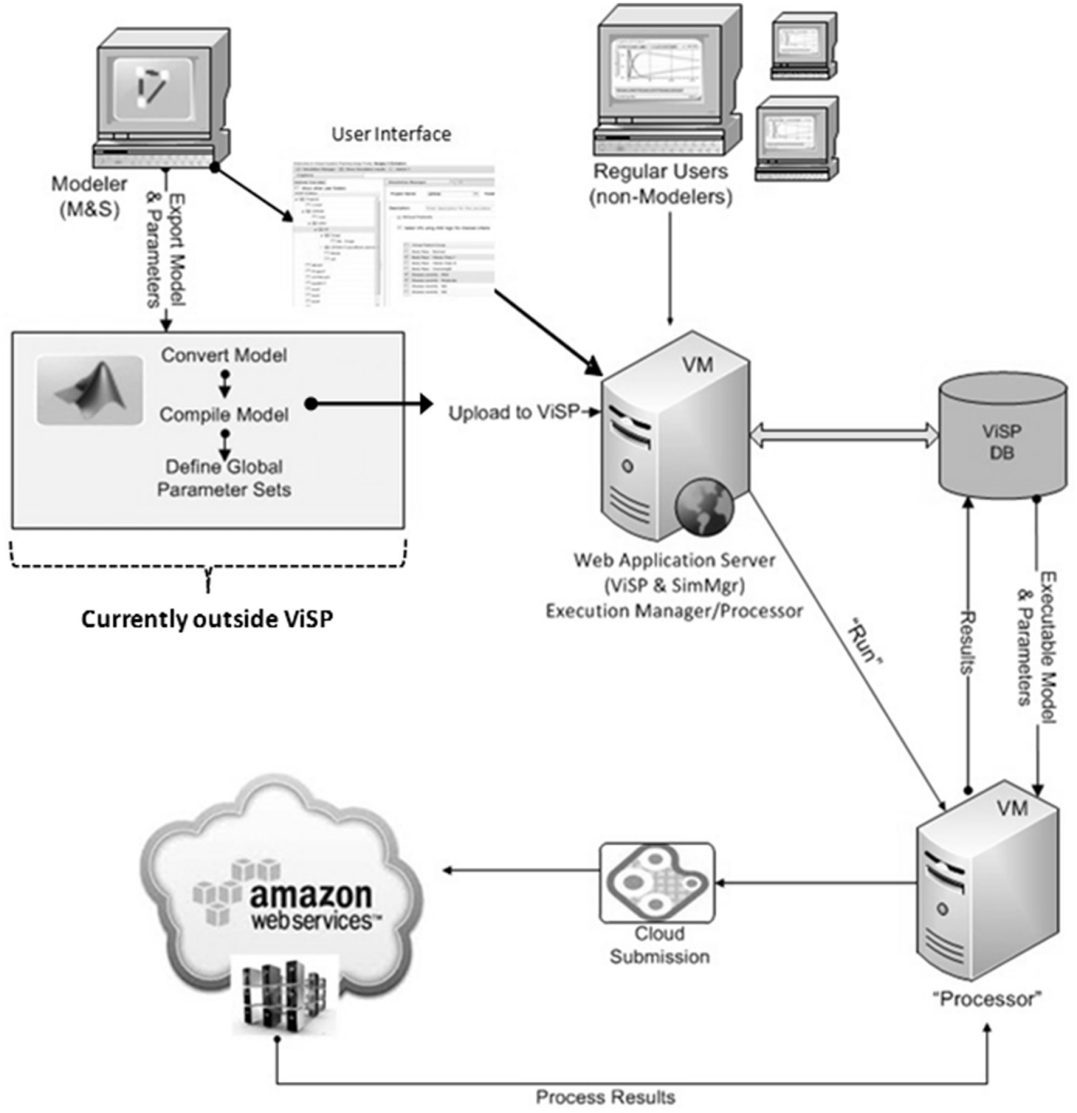
**ViSP architecture and operational diagram**. Mathematical model is implemented as a computer model by the Power User using model development software. It is then converted into an executable file with the help of 3-d party software. The Power User also configures the user interface (UI) that is specific to the model by using ViSP. Executable, text file with full set of parameter baseline values, and Virtual Patients are uploaded by ViSP into the database (DB). Power and Regular Users can then setup simulations through the UI and send them for execution. Dispatching software will distribute the simulations over the computational grid (cluster, cloud) and retrieve the results after simulations are completed. The latter will be available for analysis to users through the ViSP UI. The section of the workflow that deals with model conversion into an executable file (inside gray rectangle) is currently implemented outside of ViSP.

The software architecture with the features discussed above was implemented in the ViSP platform, a flexible tool for setting up and running large-scale QSP simulation tasks. Together with attempts to make simulation process less dependent on specific modeling tools and proprietary model formats we tried to establish a more universal workflow for simulations (Figure 2). In this article we describe the implementation of this simulation workflow using the MDSP model designed to study the effects of anti-diabetic drugs. The MDSP model itself was generated using JDesigner software (Sauro et al., 2003) and then exported as an m-file used by Matlab® software by Mathworks (MathWorks) (Figure 2). Afterwards, the “main” program controlling the simulation process was added to the model, and the code was modified such that all model parameters became input parameters as per the requirements described above. Using the Matlab Compiler Toolkit®, the code was then compiled into a standalone executable. The executable was subsequently uploaded into the ViSP database while a designated Power User (experienced user with highest privileges) configured the user interface (UI) to reflect the specifics of the model. Once the UI is in place all users may set up, submit and run simulations on any number of nodes, for instance via Amazon Web Services™. When simulations are complete the results are saved to the database in a text format for further processing. The same process can be repeated with any model as soon as it can be converted into an executable file.

ViSP specific details along with its application to the MDSP model are illustrated in the Results section. The MDSP model high-level organization is outlined in the next sub-section, however a complete description is out of this paper's scope. The mathematical basis of the MDSP model is provided in the Appendix.

### MDSP model

**Figure 3:**
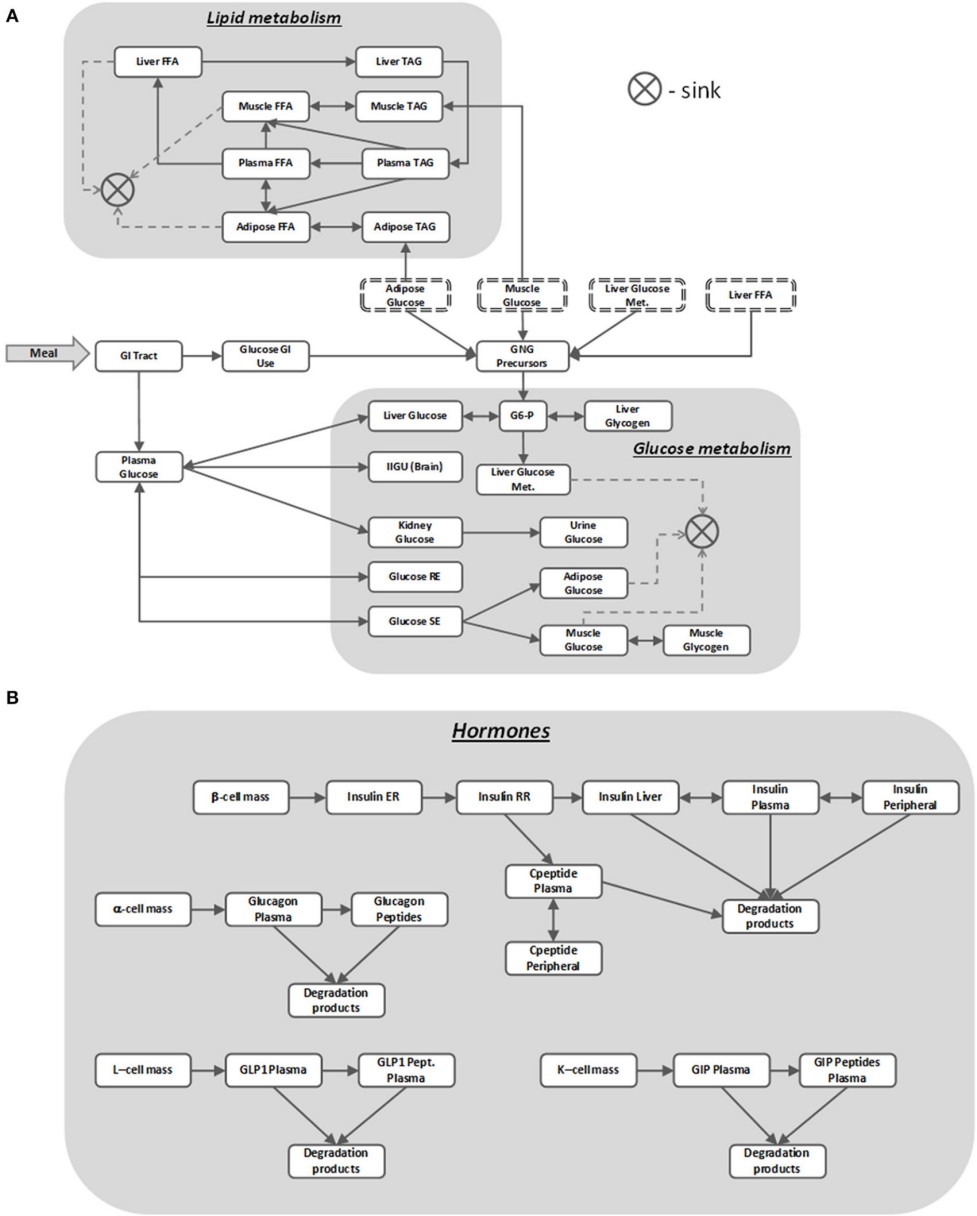
**MDSP model block diagram describing nutrients' dynamics (A) regulated by metabolic hormones (B)**. Blocks stand for dynamic quantities represented by state variables. Same name blocks with dash-line border are aliases of the blocks with solid boundaries; they are shown separately to make the diagram more readable. Arrows between the blocks point in the direction of the positive flux (reaction rate), bi-directional connectors denote either reversible flux (reaction) or two different reactions running in opposite directions. The sink element is used to remove the matter from the system that is no longer tracked, e.g., when describing glucose utilization by the muscle. For the sake of clarity not all the model details are shown on the diagram. For abbreviations used in the figure refer to the List of Abbreviations.

The MDSP mathematical model was developed to mechanistically describe the basic physiological and pathophysiological processes involved in T2DM. It represents essential systems and mechanisms regulating glucose and lipid metabolism and describes pathophysiological changes related to T2DM together with the PKPD effects for several classes of antidiabetic drugs (for recent review of mathematical models of diabetes please see Ajmera et al., 2013). The core of the model simulates intake and processing of nutrients, and their distribution and utilization by different body tissues and organs as schematically represented by a block diagram on Figure 3. The nutrients enter in the form of meals (up to three per day) with a specific percentage of carbohydrates, fats and proteins and with a given caloric content (all these can be modified through the ViSP UI). From the GI tract the nutrients are absorbed into the bloodstream and the model further tracks glucose and lipid metabolism by the brain, liver, muscle and adipose tissues (Figure 3A) (Zierler, 1999). In the liver, glucose is phosphorylated to become glucose-6 phosphate (G6-P) to be afterwards converted into glycogen (Agius, 2008). Both reactions have their counterparts working in the opposite direction such that the net glucose flux into/out of the liver maintains plasma glucose concentration within a specific range depending on the feeding condition. The liver produces glucose from the three-carbon substrates through the process of gluconeogenesis (Radziuk and Pye, 2001). The above processes are subject to insulin and glucagon regulation, and are disrupted in T2DM, resulting in increased hepatic glucose output in the postprandial and fasting states compared to healthy subjects.

Muscle also stores glucose in the form of glycogen but unlike the liver it does not use glycogen to release glucose back into circulation (Laurent et al., 2000). Insulin regulates muscle glucose uptake, storage and utilization, and T2DM have decreased sensitivity to these insulin effects. In regard to the other tissues involved in glucose metabolism, adipose tissue uptakes glucose for either storage or oxidation (Figure 3A), brain consumes glucose in a constant, insulin independent fashion, while kidneys normally reabsorb all filtered glucose unless its concentration exceeds a threshold value (Rave et al., 2006; Marsenic, 2009). Lipids in the model are represented as pools of triacylglycerols (TAGs) and free fatty acids (FFA) stored and transported between several compartments (Figure 3A).

As mentioned earlier, nutrient disposal by tissues is tightly regulated by multiple hormones, insulin being the most important one. In the MDSP model the secretion and action of insulin is described by a multistep process (Figure 3B) that is coupled to plasma glucose concentration. Other factors included in the model that affect levels of insulin are beta-cell mass and beta-cell function (Bouwens and Rooman, 2005), activation of cAMP pathways (Fridlyand et al., 2007) and activation of Ca^+^ pathways (Bertuzzi et al., 2007). Insulin is degraded primarily by the liver and partially by peripheral tissues, with C-peptide being an important by-product and biomarker of insulin secretion tracked by the model. Insulin's counterpart glucagon is described by a simpler two-compartment dynamic model (Figure 3B). Its regulatory effects are implemented as stimulating gluconeogenesis and glycogenolysis in the liver. Two other metabolic incretin hormones, glucagon like peptide-1 (GLP-1) and gastric inhibitory peptide (GIP) are also implemented in the model (Figure 3B), since they represent potential targets for therapies. GLP-1 affects glucose uptake and oxidation by adipose tissue and both hormones influence insulin and glucagon secretion in response to glucose.

The ViSP platform was used to calibrate (see Appendix) and then run the MDSP model to simulate the effects of meals, glucose and meal tolerance tests, and several antidiabetic drugs in different patient phenotypes. Examples of simulation results for two of such drugs are presented in the following Results sections. The first example illustrates the simulated effects of metformin, considered by many as a standard of care for T2DM patients, compared to literature data. The other example presents results with a relatively new class of drugs, GPR40 agonists (GPR40a), with simulations reproducing the effects of fasiglifam (TAK-875). The last example presents simulation results for metformin + TAK-875 combination therapy.

## Results

### VISP platform

**Figure 4:**
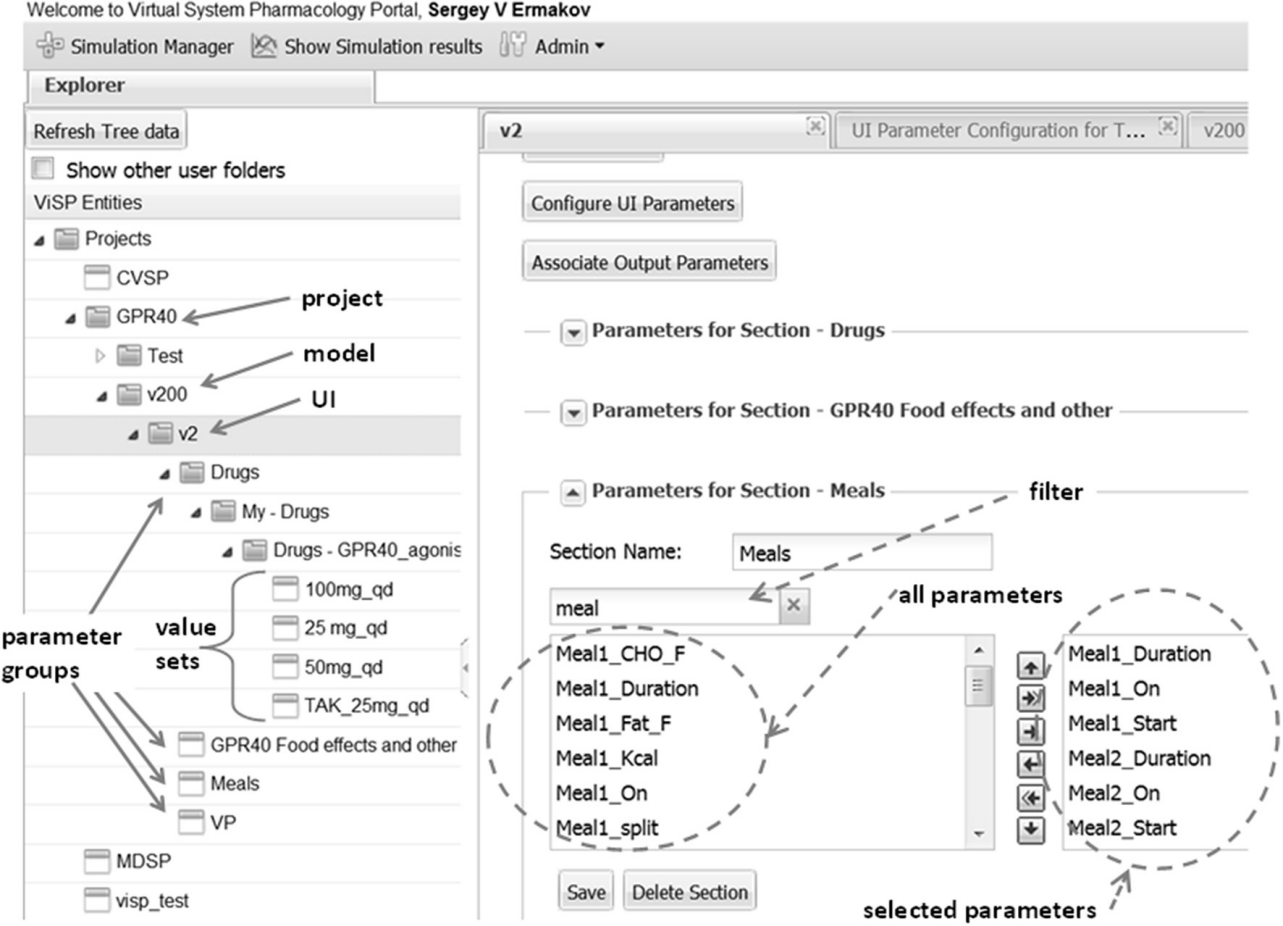
**Explorer (left side) helps to navigate through users' projects, models, and model parameters that are shown as folders**. Parameters can be organized into meaningful groups, for instance, parameters that describe a drug's regimen. The same group can be saved with different values that correspond e.g., to different doses as shown here for GPR40 agonist and can later be used for setting up simulations. Right side presents an example of defining UI section “Meals.” Circled on the left is the list box with all the parameters defined in the model that are associated with the key word “meal” provided by the user in the filter box. Outlined on the right are parameters selected to be shown on UI section “Meals.”

The ViSP software features several primary user-interface components, the first of which is the Explorer (see Figure 4, left side). It organizes user's models and data in a tree-like hierarchical structure in which top elements are projects. A ViSP project typically comprises all information related to simulation tasks that pertain to the specific research topic. Each project can contain one or several models, for instance, different versions created in the course of the model development. The model is represented by an executable file which is uploaded into the ViSP databases every time a new model is created. The executable is accompanied by a text file which contains a list of model input parameters and their baseline values. Every model can be associated with one or several user interfaces (UI) that are configured to fit particular project needs or user preferences. The next level down in the hierarchy comprises groups of parameters that are subsets of input parameters in the sense explained in the Methods. Each group may further contain multiple value sets, reflecting, for instance, settings for different drug regimens (Figure 4). All elements of the structure are stored in a database that facilitates handling of the model, data, and results.

**Figure 5:**
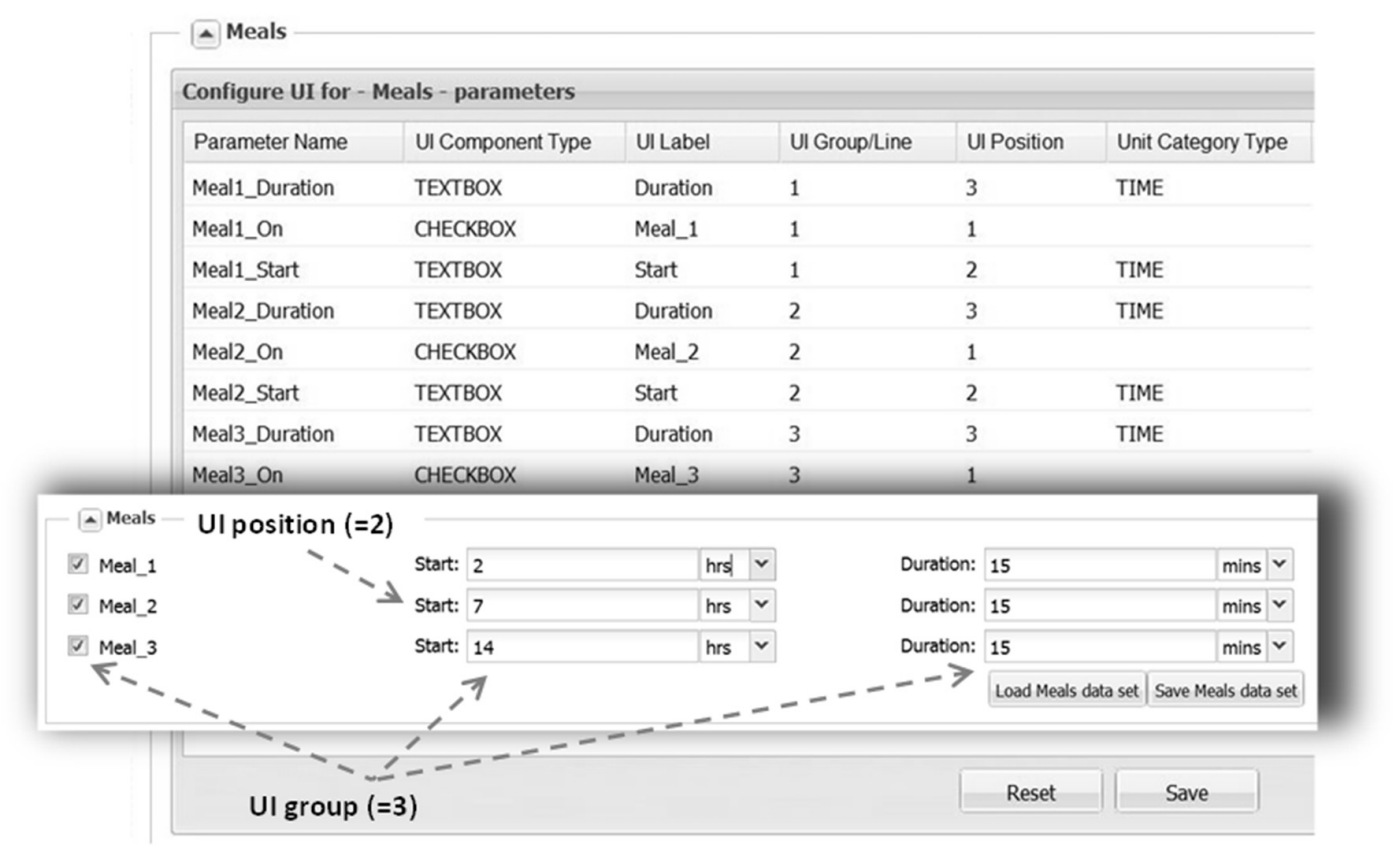
**UI layout, as shown here for the “Meals” section, is defined by filling the configuration table (background)**. Each parameter is associated with a particular control type (e.g., TEXTBOX) that is supplied with a text label. Each control location is defined by UI Group line (row) and a UI Position within the line. A parameter is also assigned a category (DOSE, TIME) in order to perform unit conversion if necessary. With the above configuration the section will look like on the inset (front).

Another important feature in ViSP is the Simulation Manager. It provides a means to customize the UI to the content of the model and then prepare and launch simulation tasks. The model UI can be configured by a Power User in a simple setup by creating sections that deal with particular aspects of the model. For example, in the section of the MDSP model specifying meal regimen, out of all parameters related to meals only the parameters defining the regimen are selected (see Figure 4, right side). Consequently, only these parameters will be presented in the UI through a series of controls. The Power User assigns meaningful text labels to these controls and specifies how they should be displayed in the UI, as a check box to turn a parameter on/off, as a text entry field, or as a dropdown selection. The configuration table helps to arrange the controls in a simple grid by specifying row and column numbers (see Figure 5). By creating various sections in this manner, the Power User has full control over which of the hundreds of parameters of the model to display, and how. The Power User can create several UIs targeting different groups of users which require particular aspects of the model to be exposed.

**Figure 6:**
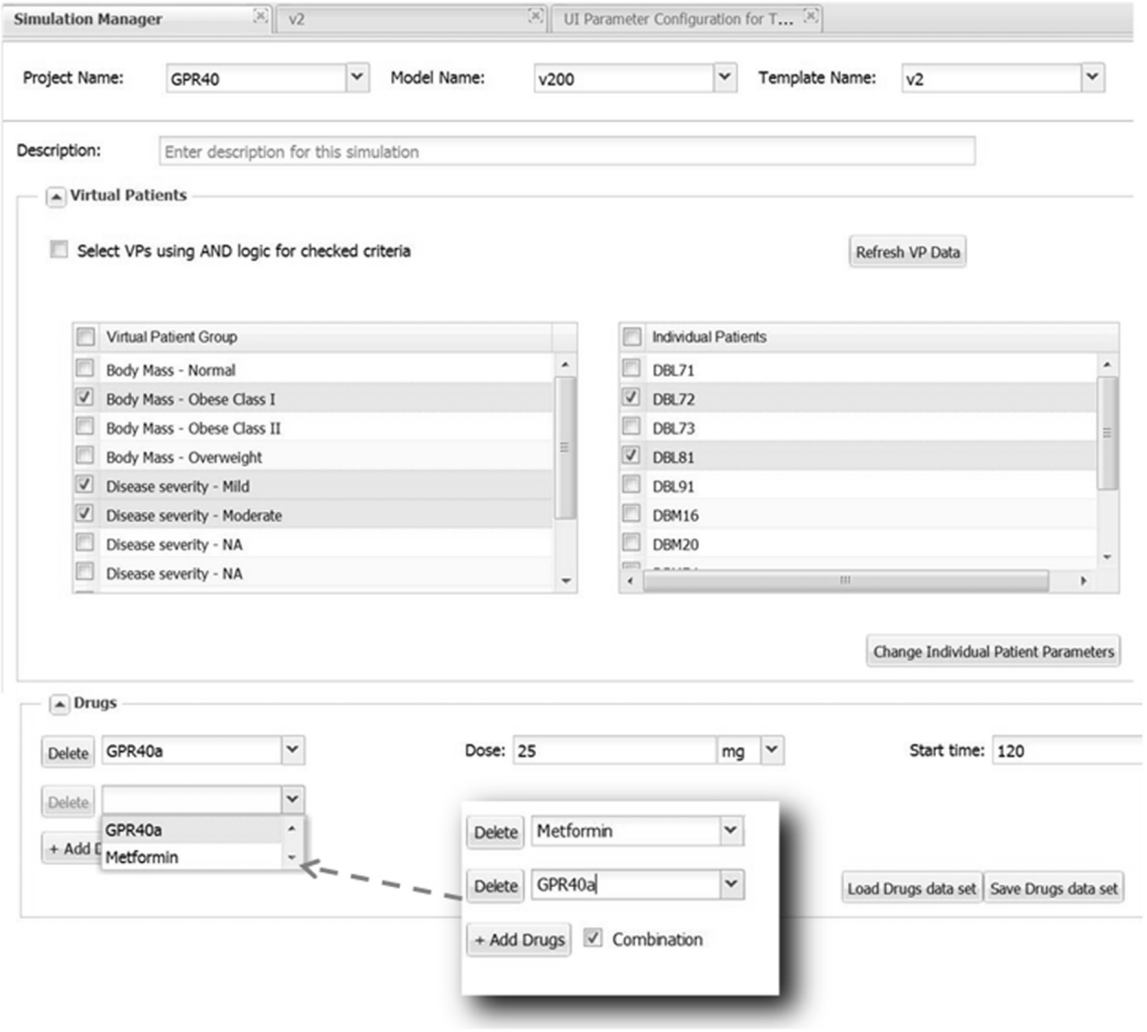
**Selecting Virtual Patients (VPs) and using parameter sections**. All available VPs are presented in the left list box inside the section “Virtual Patients” grouped according to their phenotype. By checking a box the selected phenotype will be displayed in the right list box from which a user can choose a few or all corresponding VPs to be run in simulations. Parameters for selected VPs can be modified, if necessary, by clicking the “Change Individual Patient Parameters” button that will bring up a dialog window allowing to do this (not shown). The selection of VPs belonging to multiple phenotypes could be further refined by checking and applying “AND” logic. The “Drugs” section provides an example of setting up Metformin-GPR40a combination therapy.

Once the sections of the UI have been configured, the Simulation Manager presents an option of selecting some or all of the Virtual Patients (VPs) known to the system for this model (see Figure 6). For convenience VPs are classified according to their phenotypes, thus facilitating proper VP selection required for simulations. Even though VPs come with all parameters defined, there is an option to change some of them if a user finds that necessary. After VP selection is complete, the Simulation Manager will create a set of single simulations for every combination of the settings and VPs. For example, if three VPs were selected and the parameters were set to apply one therapy, then three simulations will be generated and submitted, one for each patient. However, if two therapies were selected (the same drug with different dose, or two different drugs, etc.), then the Simulation Manager will generate six simulations accordingly. Simulation Manager also allows the therapies (dropdown selections) to be applied as “Combination” treatments, which in the above example means both therapies get applied to each patient, resulting in three simulations (see Figure 6).

The final settings that are specified through an additional UI window (not shown) are duration of simulation, output variables and time intervals between outputs. Once those are provided the simulations are fully defined and can be submitted to the computational grid. The user will get notified by e-mail upon task submission and when simulations are completed. The results that are saved in a series of text files can be retrieved afterwards via the Results Manager for further analysis and graphical visualizations. ViSP itself is capable of generating graphical plots which can be viewed directly as part of the results.

ViSP's Administrator tool provides means to register and grant access only to users who are authorized to use the software and its data, thus preventing any proprietary information from disclosure. Additionally all users are divided into Power Users and Regular Users based on their privileges. As was described above Power Users are allowed to create and modify projects, configure model UIs, and set up and run simulations, while Regular Users can perform only the last two functions.

### Simulations metformin

**Figure 7:**
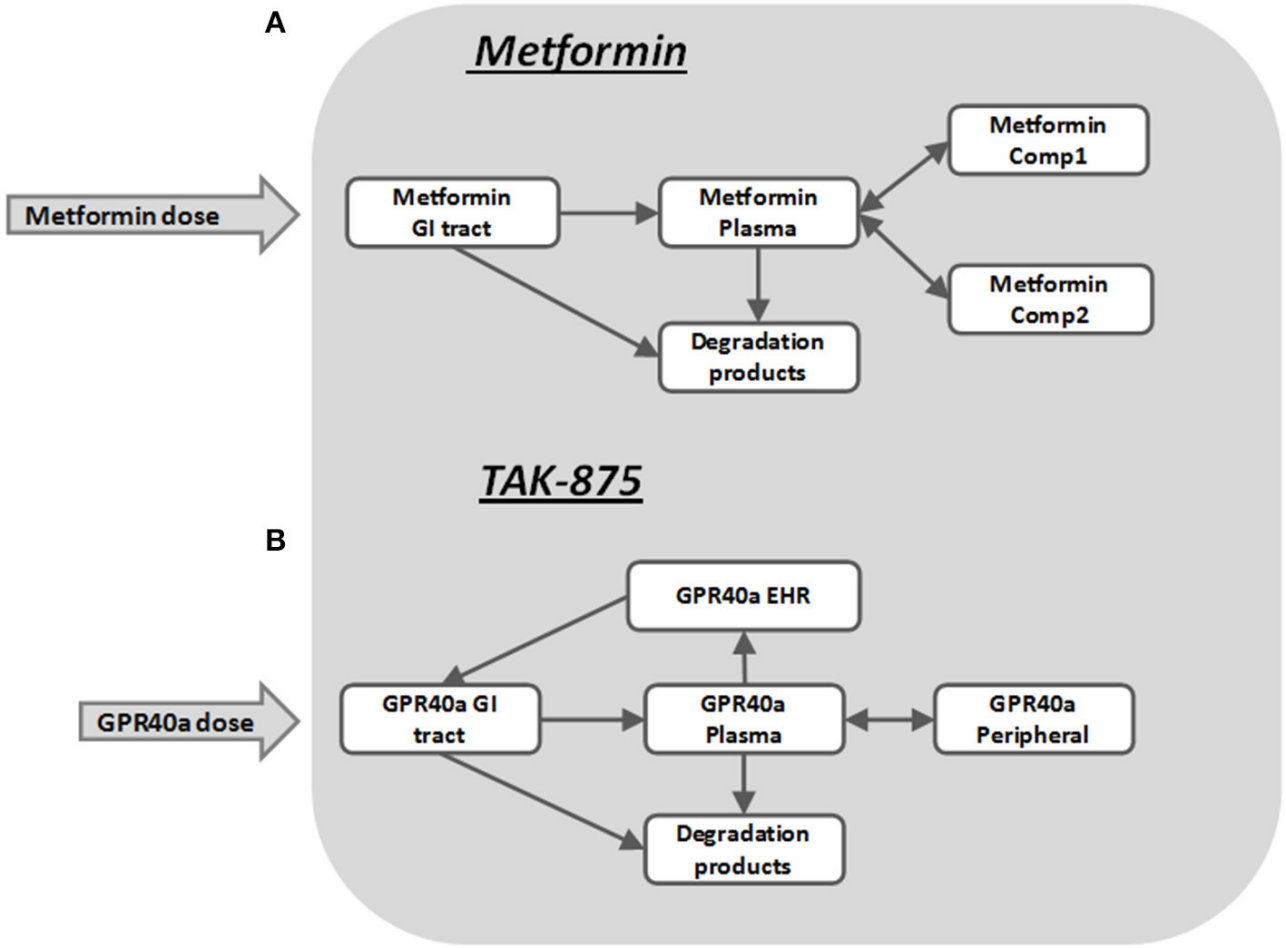
**Pharmacokinetic sub-models for Metformin (A) and GPR40 agonist TAK-875 (B)**.

**Figure 8:**
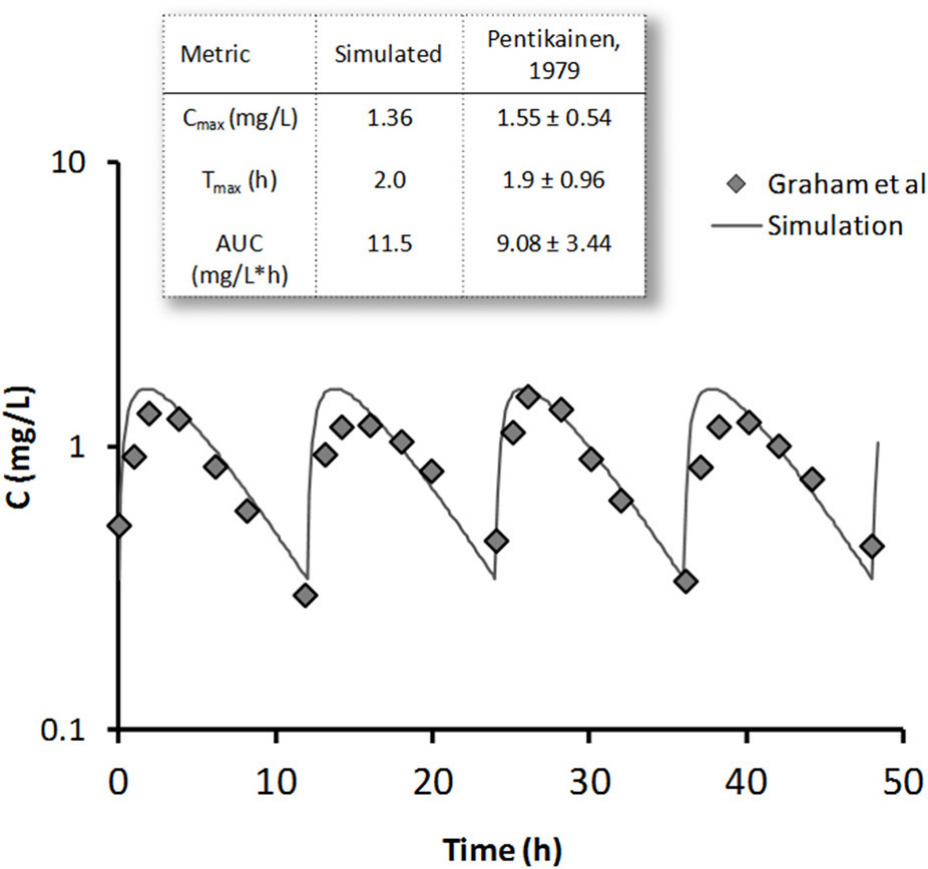
**Metformin simulated steady-state concentration profile (line) is plotted against clinical data (diamonds) (Graham et al., 2011) for 500 mg bid dose regimen**. On the inset single 500 mg dose simulation results are compared with data from Pentikainen et al. (1979) (mean ± SE) for metformin peak concentration *C*_max_, time to peak *T*_max_, and area under the curve (AUC).

The pharmacokinetics of metformin was simulated by using a three-compartment model (Figure 7A) derived from the Pentikainen et al. (1979). The model was calibrated to fit PK characteristics for a 500 mg single dose (Pentikainen et al., 1979) and multiple 500 mg twice daily doses (Graham et al., 2011) obtained with healthy individuals. An adequate fit has been achieved for both data sets as evidenced by Figure 8. It was deemed acceptable to apply the same calibration for simulating metformin pharmacodynamic (PD) effects in T2DM patients. This assumption is supported by the data from the Tucker et al. (1981), which found little difference in metformin PK between healthy and T2DM individuals.

Metformin has multiple sites of action, including liver, muscle, adipose tissue, GI tract, and pancreas. Despite the fact that metformin is perhaps the most widely used antidiabetic therapy its exact mechanism of action remains unclear (Kirpichnikov et al., 2002). Among its reported primary PD effects are decreased hepatic glucose production (Stumvoll et al., 1995; Campbell et al., 1996), increased peripheral tissue sensitivity to insulin (Bailey and Turner, 1996), and increased glucose utilization. Other less commonly described effects include lowering FFA levels and increasing lipid oxidation (Perriello et al., 1994), increased glucose utilization by the GI tract, and a delayed, more distal GI glucose absorption (Bailey et al., 2008). There are different opinions on whether metformin directly affects β-cells (DeFronzo, 1999), however some evidence exists that it improves the function of β-cells (Patane et al., 2000; Bi et al., 2013) and their response to glucose. In the MDSP model the above mentioned metformin PD effects were implemented as multipliers in the rate equations. Functionally they are expressed as Hill equations (Appendix, Equation A3) representing either metformin-mediated activation or inhibition.

A study on the short-term effects of metformin in T2DM patients (Eriksson et al., 2007) was selected for simulations demonstrating the model's ability to reproduce metformin therapeutic outcomes. In this study an escalating metformin dose (500 mg *qd* for 7 days followed by 500 mg *bid* for 7 days and then by 1000 mg *bid* for 14 days) was applied to a group of T2DM patients with fasting plasma glucose concentration between 7 and 12 mM. At the beginning of each subsequent dose an oral glucose tolerance test (OGTT) was performed to check the effects of the previous dose on glucose and other metabolic characteristics, (for further details see paper by Eriksson et al., 2007). In simulations the same treatment regimen was reproduced for a representative virtual patient that matched the study enrollment criteria including body weight, fasting plasma glucose (FPG), age, etc. Table 1 provides a comparison between clinical and simulation data for key parameters, including FPG, area under the glucose concentration curve for OGTT, and percent change in fasting plasma insulin (FPI) concentration from day 0 before treatment and after 7, 14, and 28 days of metformin. Overall there is good agreement between simulations and data, with simulations slightly under-predicting the decrease in FPG especially at higher doses.

**Table 1:** **Effects of metformin in 28 day study in T2DM patients, comparison between clinical data by Eriksson et al. (geometric mean ± 95% conf. interval) and simulation**.

**Days of treatment**	**0**		**7**		**14**		**28**	
**Data metric**	**Eriksson et al**.	**Sim**.	**Eriksson et al**.	**Sim**.	**Eriksson et al**.	**Sim**.	**Eriksson et al**.	**Sim**.
FPG (mM)	9.54 (8.6, 10.6)	10.1	9.15 (8.24, 10.15)	10.1	8.26 (7.55, 9.46)	9.6	7.59 (6.74, 8.54)	8.6
AUC glucose (mM·h)	29.6 (27.1, 32.4)	29.9	26.4 (24.2, 28.7)	24.9	23.4 (21.5, 25.5)	20.5	21.7 (19.9, 23.6)	19.9
FPI (% change from day 0)	0 (–29, 41)	0	5 (–23, 43)	2	16 (–42, 23)	16.0	1 (–24, 29)	8.0

### GPR40 agonist (TAK-875)

**Figure 9:**
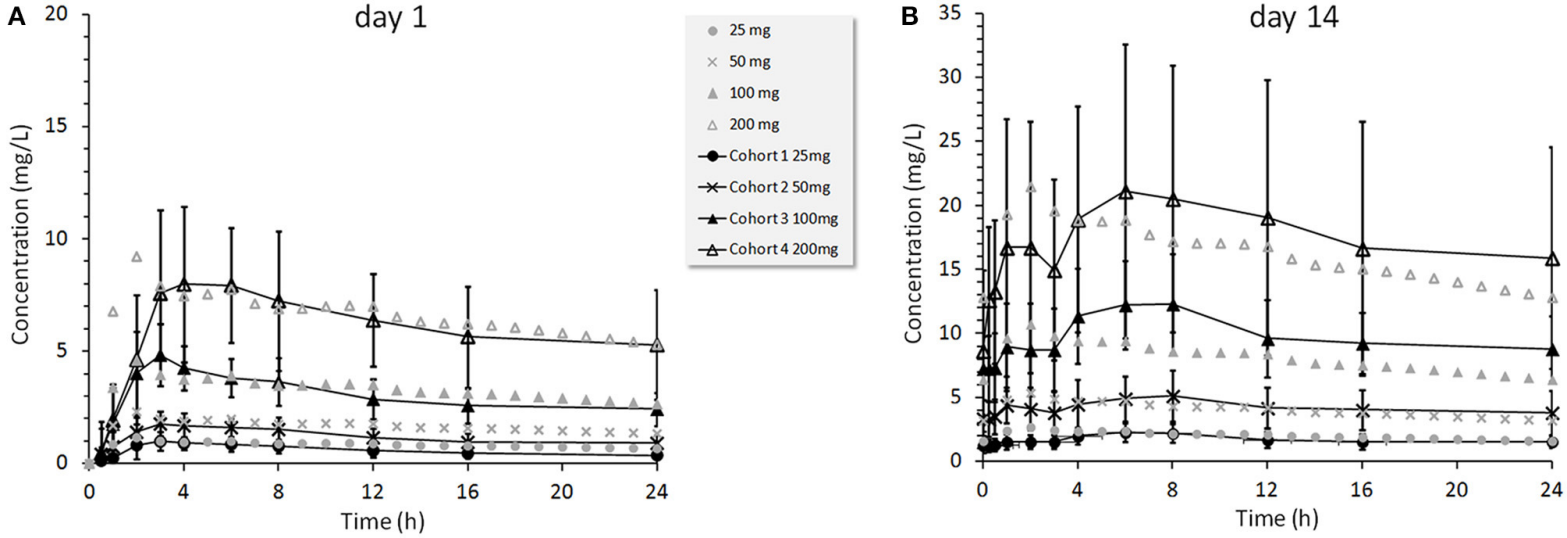
**TAK-875 concentration profiles for 25, 50, 100, and 200 mg once daily doses administered to T2DM patients**. Clinical data points are mean values ±SD (Leifke et al., 2012) shown by black color markers connected with lines overlaid with simulated data shown by gray color markers only. Panel **(A)** presents results at day 1, panel **(B)** at day 14.

TAK-875 is a selective GPR40 agonist that improves glycemic control in T2DM patients by potentiating postprandial insulin secretion in a glucose dependent manner with a minimal risk for hypoglycemia (Kaku, 2013; Yabuki et al., 2013). A single dose TAK-875 PK study with healthy volunteers (Naik et al., 2012) and a multiple dose study with T2DM patients (Leifke et al., 2012) were used to establish and calibrate the PK section of the MDSP model (Figure 7B). An enterohepatic recirculation (EHRC) was included in order to better fit the TAK-875 clinical data (Figure 9).

The mechanism by which TAK-875 potentiates insulin secretion involves activation of GPR40 in pancreatic β-cells followed by a cascade of reactions increasing the levels of secondary intracellular messengers. This eventually results in increased Ca^2+^ release that enhances the movement of insulin granules and their fusion with the plasma membrane, leading to subsequent insulin release (Burant, 2013). In the MDSP model all these events are simplified into one mechanism representing the net TAK-875 amplification of the Ca^2+^ effect on insulin secretion. GPR40 is also expressed in enteroendocrine cells of the intestine, and it has been hypothesized that GPR40 activation may potentially lead to increased secretion of GLP-1 and GIP hormones (Luo et al., 2012; Mancini and Poitout, 2013). These pathways are represented in the model as hypotheses, so that their potential impact on efficacy could be evaluated. However, the results of the TAK-875 clinical study in T2DM patients did not demonstrate increases in GIP or GLP-1 following an OGTT (Leifke et al., 2012). Therefore, the secretion of GIP and GLP-1 via the intestine was disabled for simulations with TAK-875. In choosing a representative VP for simulations, as in the case of metformin, we selected one with steady-state characteristics comparable to the mean values found in the study enrollment criteria.

**Figure 10:**
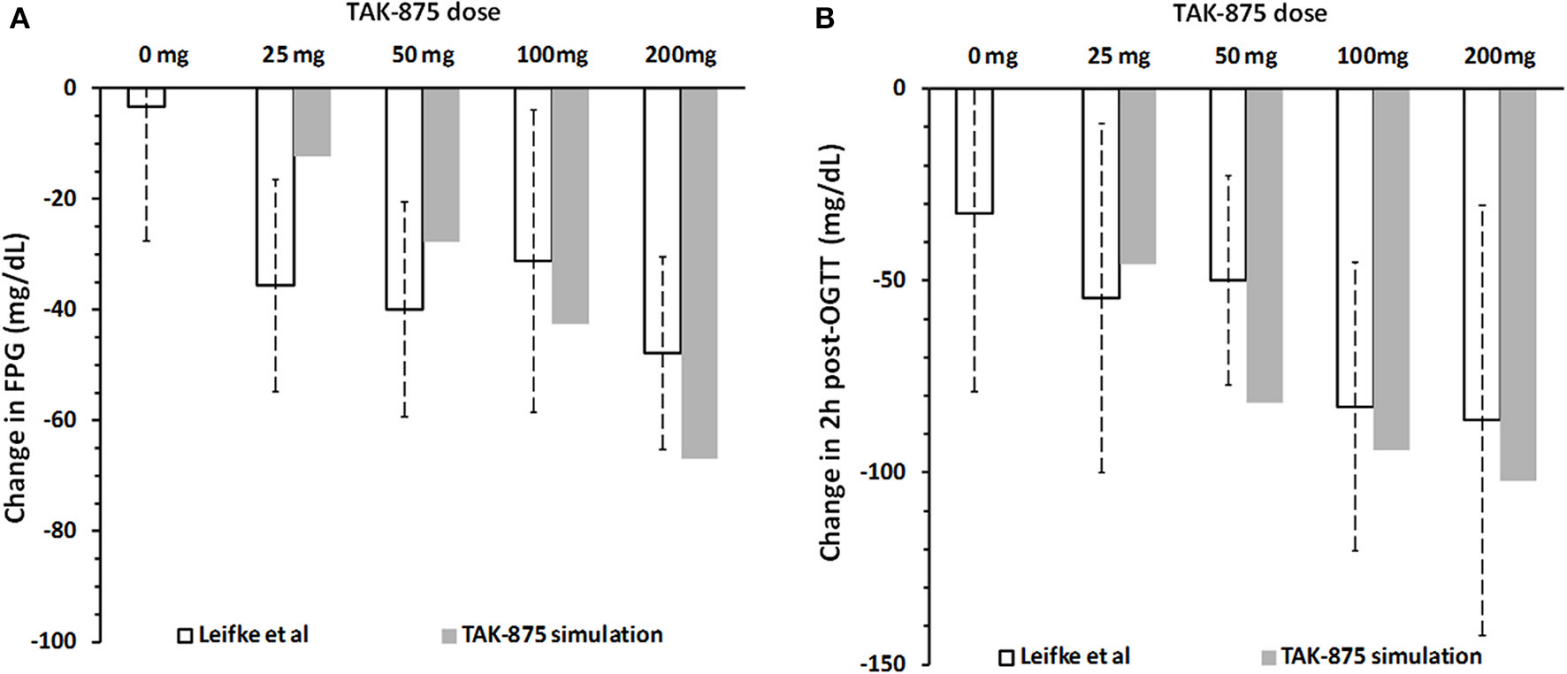
**Change in fasting plasma glucose concentration (A) and 2 h post-OGTT glucose values (B) from baseline after 14 days of placebo or 25, 50, 100, and 200 mg daily dose of TAK-875**. Panels **(A,B)** show clinical data (Leifke et al., 2012), and simulation results for TAK-875 monotherapy.

Figure 10 compares simulation results with clinical data from a multiple ascending dose study of TAK-875 in T2DM subjects that received either placebo or one of the 25, 50, 100, 200 mg daily doses (Leifke et al., 2012). Data shown in the figure illustrate the short-term TAK-875 effects on steady state responses (FPG concentration, Figure 10A), and dynamic responses (2 h post-OGTT glucose concentration Figure 10B) after 14 days with different levels of drug exposure. Simulations provide adequate predictions in both occasions although simulated glucose post-OGTT values seem to follow clinical data more closely.

### Metformin—TAK875 combo

**Figure 11:**
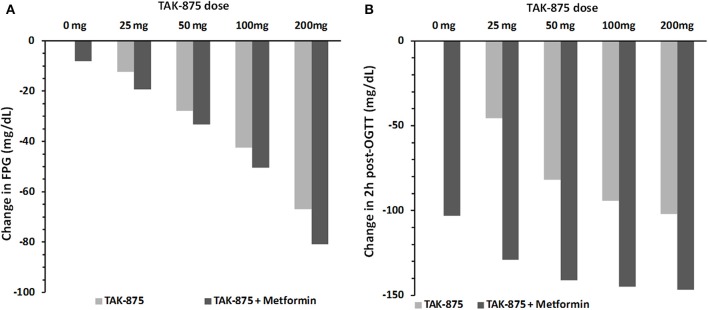
**Simulated change in fasting plasma glucose concentration (A) and 2 h post-OGTT glucose values (B) from baseline after 14 days of placebo or 25, 50, 100, and 200 mg daily dose of TAK-875**. Panels **(A,B)** compare simulation results for TAK-875 monotherapy with TAK-875 + 500 mg bid metformin combo.

Since metformin is used as the standard of care for treating hyperglycemia in T2DM patients, we repeated the above simulation of TAK-875 in combination with 500 mg metformin twice daily as a background therapy. Simulation results suggest that additional therapeutic benefits could be achieved by combination therapy by further lowering FPG and post-prandial glucose excursions (Figure 11). Interestingly, the effect on post-prandial glucose appears to be more pronounced, with the response at TAK-875 doses of 50 mg and higher approaching a plateau (Figure 11B). In contrast the decline in FPG over the same dose range (Figure 11A) did not appear to have reached saturation.Without metformin (Figure 10) this plateau in OGTT response is observed in both clinical and simulation data but at higher (>100 mg) doses than with combination therapy.

## Discussion

QSP models bring new insights into our understanding of the mechanism of action of drugs and they help in optimizing decision making in pharmaceutical R&D (Schmidt et al., 2013). However, multiple obstacles need to be overcome in order to increase recognition of the value of QSP within the pharmaceutical industry. Two challenges are worth mentioning in the context of this article. First, QSP models rely on large-scale simulations requiring high-performance parallel computing infrastructure that is expensive to run and maintain. Second, there is no industry standard software, such as NONMEM® for non-linear mixed effects PK/PD modeling that satisfies the diverse needs of the modeling community. Utilizing multiple tools increases the cost of model development and limits the exchange of models between scientists, thus creating additional barriers for model acceptance and application. A solution for the first challenge could be cloud-based computing, as the burden of creating and maintaining an up to date computational environment is outsourced to vendors of high-performance computing clusters (e.g., Amazon). By developing the ViSP platform we attempted to address the second challenge, i.e., making the simulation process less dependent on the modeling tools and creating a more universal workflow for simulations (Figure 2). The central idea behind the ViSP platform is to work with the model file once it satisfies two conditions; first, it is compiled into an executable file, and second, all model parameters are presented as input parameters. Combination of an executable binary file with an input text file fully defines a single simulation task that has the following benefits. On the one hand, it is no longer dependent on the file format or the specifics of the modeling tool that created the model. On the other hand, the model preserves all possibilities for its customization since all its parameters are available through the input file. Additionally, when launched it runs as a single computer process that provides flexibility in choosing the hardware (multicore processors, cluster, or cloud) that can be used for computations. The only requirement here is to use the proper compiler when creating the executable file.

Large-scale simulation tasks in which ViSP could be useful originate from numerous applications. We employed ViSP for calibrating the MDSP model and for simulating clinical studies. In the first case, multiple virtual patients (VPs) have been created in which only the parameters of interest were changed. Then a series of simulations equal to the number of virtual patients has been run and the process was repeated until the desired model behavior was achieved. The latter meant checking that sets of output parameters lay within the observed ranges derived from clinical or preclinical data. A similar procedure was used to create new VPs representing different phenotypes. In the future we are planning to automate this process, when parameter variations and model response analysis will be done without user intervention. The process just described could be applied to perform sensitivity analysis, for instance, to search for the pathway that responds the most (or the least) to the drug, or to characterize the drug response based on patient phenotype. This process could also be used to model a clinical trial, when different cohorts of VPs that satisfy the enrollment criteria are simulated and their responses are analyzed to provide suggestions for patient stratification.

One aspect of simulation workflow that remains outside the capabilities of the ViSP platform is how to convert model files saved in proprietary formats into an executable code (see Figure 2). Currently there is no universal mechanism inside ViSP allowing this to be done with an arbitrary file format. The proprietary nature of the model files prevents seeing model details, such as equations and parameters, making compiling such files into an executable impossible. Normally the modeling software itself does not offer this option either. The solution, however, exists if the modeling tool allows the export of models into a file format that can be read by other software. One such format is Systems Biology Markup Language (SBML), a computer language that is gaining ground inside the Systems Biology community for saving and exchanging models between users. Currently several model development tools offer SBML export capabilities, among them JDesigner (part of the Systems Biology Workbench, SBW) (Sauro et al., 2003), SimBiology by Mathworks (MathWorks), CellDesigner by the Garuda Alliance (Kitano et al., 2005), DBSolve Optimum by ISB (Gizzatkulov et al., 2010) and others. Once saved in SBML format, a model file can be translated into a different computer language that afterwards can be compiled into an executable (see Figure 2). We utilized SBW capabilities to export an SBML file into a MathWorks Matlab® file that later was compiled into a binary executable file using the MathWorks compiler. Since the MDSP model was originally developed in JDesigner, which “natively” saves models in SBML (XML) format, there were no issues in exporting it to a Matlab file. However, if the SBML model file is produced by a different modeling tool, for example PhysioLab®, it may require some editing before saving it as Matlab code. Such modifications may be necessary since the level of SBML support varies in different modeling tools.

In conclusion, we developed a versatile web based software platform that provides capabilities for setting up and running massive simulation tasks originating from system-level mechanistic models. It is designed to conveniently handle diverse modeling projects with large number of parameters while being flexible with respect to model structure. Its utility was demonstrated with metabolic diseases model by simulating pharmacological effects of antidiabetic drugs, metformin and fasiglifam in healthy and diabetic individuals.

### Conflict of interest statement

Sergey Ermakov, Jyotsna Pagidala, Marko Miladinov, Albert Wang, and Tarek A. Leil are employees of Bristol-Myers Squibb, P.Forster is an employee of Forster Solutions, LLC, Rebecca Baillie, Derek Bartlett, and Mike Reed are employees of Rosa and Co. LLC.

## Footnotes

^1^http://www.mathworks.com/products/simbiology/.

## Appendix

### Basic equations

Mathematically the MDSP model is organized as follows. Individual blocks *i* (*i* = 1, …, *n*) shown on Figure 3 correspond to quantities of a material *Q*_*i*_ (e.g., amount of nutrient, number of cells, etc.) distributed within a particular compartment. Blocks may also stand for a state or a signal attributed to an organ or body system, like e.g., vagal stimulation of the stomach. The quantities are expressed as time-dependent state variables described by ordinary differential equations (ODEs) (Equation A1) where the right side term *F*_*i,j*_ signifies the flux of *Q*_*j*_ to compartment *i* (shown by arrows on the diagram). These fluxes may depend on multiple variables, for instance, concentration of hormones and/or drugs. Most of them follow similar functional form (Equation A2) in which basal rate *k*_*i,j*_ is modified by activation, represented by the term α_*i,j*_ and/or by inhibition, represented by the term β_*i,j*_. Activation and inhibition terms are given by either the Hill-type equation (Equation A3) or by a sigmoid shape function (Equation A4) where coefficients γ_*i,j*_ and σ_*i,j*_ regulate the slope and the shift of the curve. Here functions are shown for activation term α_*i,j*_, they are identical for β_*i,j*_. Parameters α^*max*^_*i,j*_ may assume only non-negative values. In the case of inhibition, β^*max*^_*i,j*_ are further restricted to be no greater than 1. The variable *P*_*j*_ may stand for either the quantity *Q*_*j*_ or its concentration *C*_*j*_ in case when *Q*_*j*_ represents the amount of substance. Parameters *n*_*i,j*_, γ_*i,j*_, σ_*i,j*_ are constant values. In each particular instance the choice of the function, either Equation A3 or Equation A4, is dictated by a prior knowledge about the mechanism of action. Alternatively it is selected based on which function provides better fitting to available data. For details refer also to Figure 3.

(A1) $$\frac{dQ_{i}}{dt}=\sum_{j}F_{i,j}$$

(A2) $$F_{i,j}=k_{i,j}\;\cdot \;Q_{j}\;\cdot \;\left(1+\alpha_{i,j}\right)\left(1-\beta_{i,j}\right)$$

(A3) $$\alpha_{i,j}=\alpha_{i,j}^{max}\;\cdot \;\frac{P_{j}^{n_{i,j}}}{P_{j}^{n_{i,j}}+K_{j}^{n_{i,j}}}$$

(A4) $$a_{i,j}=\alpha_{max}\;\cdot \;\frac{1}{2}\left(1+tanh\;\left(\gamma_{i,j}\left(P_{j}-\sigma_{i,j}\right)\right)\right)$$

In addition to ODEs, the MDSP model contains a number of algebraic equations that calculate additional quantities and parameters, for instance, insulin sensitivity index QUICKI (Katz et al., 2000), homeostatic model assessment index HOMA (Wallace et al., 2004) and others. Overall the model comprises more than 100 ODEs and more than 50 algebraic equations resulting in a large number of parameters (more than 800) associated with them.

### Model calibration

As explained in the Methods section, parameters in the model could be approximately divided into those describing the characteristics of an individual subject [virtual patient (VP)], parameters that represent drugs, parameters for clinical interventions, e.g., OGTT, parameters that describe meal regimen, and the like. All of them are input data defining a particular simulation. Whenever possible the values for parameters are taken from the literature, however, when the values are not available they are estimated by matching model behavior to known clinical and preclinical data. For a valid VP, values for plasma concentration of glucose, insulin, C-protein, glucagon, GLP-1, GIP, FFA, TAG obtained after simulated overnight fast are required to match clinical data characteristic of the phenotype this VP represents (healthy or T2DM). In addition a valid VP should correctly reproduce simulated interventions such as a standard OGTT and meal tolerance test (MTT) with glucose and insulin concentration profiles being similar to the ones observed clinically. Since multiple sets of parameters could potentially match the same data, these sets constitute alternative VPs that reflect clinical variability. Parameters defining VPs can be varied intentionally to produce subjects representing different behaviors (phenotypes). For the MDSP model we have created a number of VPs that we classified as belonging to the following 5 phenotypes: normal healthy, obese non-diabetic, type 2 diabetic patients with mild, moderate, and severe degree of the disease. Each VP is defined by more than 800 parameters and ViSP provides the convenience of storing and handling them in a structured way.

In general the simulations were performed as follows. At first all VPs are simulated for a period of time until they reach a steady state with a meal regimen that matches VP energy expenditure. The latter is calculated based on VP age, height, body weight, and activity level. After the steady state is achieved the simulation of interest is initiated by applying additional sets of parameters corresponding to drugs and interventions that reproduce the conditions of the clinical trial or other experiments of interest.

## Abbreviations

ER: extended release
FFA: free fatty acids
G6-P: glucose-6 phosphate
GI: gastrointestinal
GNG: gluconeogenesis
GIP: gastric inhibitory peptide
GLP-1: glucagon like peptide 1
GPR40: G-protein coupled receptor 40
IIGU: insulin independent glucose utilization
MDSP: metabolic diseases systems pharmacology
PD: Pharmacodynamic
PK: pharmacokinetic
RE: rapidly equilibrating
RR: readily releasable
SE: slowly equilibrating
TAG: triacylglycerol
UI: User Interface
ViSP: virtual systems pharmacology.
